# Next-Generation Sequencing in Early Diagnosis of Dent Disease 1: Two Case Reports

**DOI:** 10.3389/fmed.2018.00347

**Published:** 2018-12-07

**Authors:** Min Wen, Tian Shen, Ying Wang, Yongzhen Li, Xiaoliu Shi, Xiqiang Dang

**Affiliations:** ^1^Department of Pediatrics, The Second Xiangya Hospital, Central South University, Changsha, China; ^2^Laboratory of Pediatric Nephrology, Institute of Pediatrics, Central South University, Changsha, China; ^3^Department of Medical Genetics, The Second Xiangya Hospital of Central South University, Changsha, China

**Keywords:** dent disease 1, next-generation sequencing, low molecular weight proteinuria, *CLCN5* gene mutation, early diagnosis

## Abstract

Dent disease 1 is a rare X-linked recessive inherited disease, caused by pathogenic variants in the chloride voltage-gated channel 5 (*CLCN5*) gene. Dent disease 1 is characterized by low molecular weight (LMW) proteinuria, hypercalciuria, nephrocalcinosis, and chronic kidney disease. Infants may manifest only asymptomatic LMW proteinuria, which increases the difficulty of early diagnosis. We describe two male infants presenting only with nephrotic-range LMW proteinuria observed on examination using urine protein electrophoresis. Hereditary renal tubular diseases were highly suspected based on early onset age and LMW proteinuria. Thus, next-generation sequencing (NGS) was performed and pathogenic mutations in *CLCN5* were identified in both patients. A diagnosis of Dent disease 1 was established based on the above informations. The two patients developed hypercalciuria during late follow-up, which verified the diagnosis. These two cases highlight the importance of next-generation sequencing in the early diagnosis of Dent disease 1 with only LMW proteinuria.

## Introduction

Dent disease 1 (OMIM 300009) is a proximal renal tubular dysfunction that occurs mostly in males. It is a rare X-linked recessive inherited disease, caused by pathogenic variants in the *CLCN5* gene. The diagnosis of Dent disease 1 (1, 2) is based on the presence of all three of the following characteristics: (1) LMW proteinuria [Commonly retinol binding protein (RBP) and α1-microglobulin]; (2) hypercalciuria; and (3) at least one of the following: nephrocalcinosis, nephrolithiasis, hematuria, hypophosphatemia, or renal insufficiency. It is important to note that Dent disease 2 (OMIM 300555), caused by *OCRL* mutations, shares the same diagnostic criteria. Like many other inherited diseases, the prognosis of Dent disease 1 is poor. Dent disease 1 accounts for about two-thirds of all cases of Dent disease (3) and 30–80% of affected males develop end-stage renal disease (ESRD) between the ages of 30 and 50 years (1). Thus, early diagnosis is critical for the prognosis.

However, early diagnosis of Dent disease 1 in routine clinic work is difficult. Szczepanska et al. (4) conducted a retrospective clinical and genetic analysis of 10 unrelated patients with Dent disease in Poland, of which 9 patients carried a mutation in the CLCN5 gene. The earliest age of diagnosis was 5 years when the patient showed obvious proteinuria and hypercalciuria. The onset of symptoms of Dent disease 1 may appear at an early age; however, in many reported Dent disease 1 cases (5–7), the age of diagnosis is rather late. Young patients always present with only proteinuria at onset, even nephrotic-range proteinuria, which makes the early diagnosis of Dent disease 1 even more difficult. van Berkel (8) summarized the information of 148 patients from 47 reports, finding that more than half of the patients with Dent disease have nephrotic range proteinuria. One case (9) was initially misdiagnosed as nephrotic syndrome so that corticosteroids and immunosuppressive therapy (including cyclophosphamide and mycophenolate mofetil) were incorrectly used for treatment. Another problem is that urine protein electrophoresis has not been routinely used for screening for LMW proteinuria (10), thus, missing many opportunities to find tubular disease. LMW proteinuria can be found in a number of genetic tubulointerstitial diseases, such as nephronophthisis, Dent disease 1, Dent disease 2, Lowe syndrome, and cystinosis, in addition to the acquired diseases (11), which need next-generation sequencing (NGS) to be diagnosed. Therefore, insufficient awareness of genetic analysis is also an important factor that limits early diagnosis.

In this report, we describe two male infants who presented with only the symptom of nephrotic-range LMW proteinuria and in whom NGS was used in the early diagnosis of Dent disease 1.

## Cases

### Case 1

A male infant was examined for nephrotic-range proteinuria during hospitalization for pneumonia after birth. Other symptoms, such as edema, gross hematuria, hypertension, and hypoproteinemia were not observed. He was brought to our hospital for diagnosis and treatment when he was 9 months old. At that time, urine routine test still showed protein 3+, and serum albumin was 39.3 g/l with a normal renal function. Other laboratory tests and urinary tract ultrasonography showed no obvious abnormalities. Pregnancy and birth were normal and term. His maternal grandparents are cousins, without any indication of renal disease. We examined the urine protein by electrophoresis analysis, which showed LMW proteins, RBP, α1-microglobulin and β2-microglobulin as the main components (Table 1). Due to the nephrotic-range proteinuria for 9 months, renal biopsy was done and showed focal segmental glomerular sclerosis(FSGS). After holding written informed consent, the child and his mother underwent whole-exome sequencing to screen for hereditary tubular disease. The exon of *CLCN5* gene (ChrX:49834680) found a hemizygotic mutation (c. C310>T,p.R104X) in the child. The same mutation site was reported in another Dent disease 1 case (12), who showed hematuria, proteinuria, hypercalciuria, renal phosphate wasting, aminoaciduria, β2-microglobulinuria, and active Rickets at 2 years old. The gene mutation was not detected in his mother, implying that the child carries a *de novo* mutation. At 5 months follow-up, the child developed hypercalciuria with calcium to creatinine ratio 0.628 in a spot sample [normal range <0.81(0-1Y), <0.56(1-2Y) (1)] and was administered hydrochlorothiazide for the treatment.

**Table 1:** Clinical parameters.

**Patient**	**Case 1**	**Case 2**	**Normal range**
Urine protein:creatinine ratio	0.925	/	<0.2
Urine calcium:creatinine ratio	0.168	0.236	<0.81(0-1Y), <0.56(1-2Y)
Twenty-four-hour protein excretion	/	495.02	<150
Urine erythrocyte (/hpf)	<8000	<8000	<8000
Urine microalbumin (mg/l)	112.36	708.15	<20
Urine α1-globulin (%)	165.23	919.5	<12
Urine β2-globulin (mg/l)	118.8	838.47	0.10-0.30
Urine retinol binding protein (mg/l)	75.1	481.27	<0.70
Urine N-acetyl-β-D-glucosaminidase (u/l)	29.51	163.82	0.30-11.50
Nephrocalcinosis	No	No	No
Genetic analysis	*CLCN*5 (c.C310T, p.R104X)	*CLCN*5 (c. A815G, p.Y272C)	No
FSGS	Yes	/	No

“/” *means not tested; “No” shows normal items; “FSGS” means focal segmental glomerulosclerosis*.

### Case 2

A routine urine test of a 19-month-old male infant showed urine protein ++ and occult blood. Twenty-four-hour protein excretion was 1340 mg/24 h. Serum albumin was 39.3 g/l. Twenty days later, he was sent to our hospital for diagnosis and his urine protein and occult blood were tested in our ward; results were still positive. No other symptoms and abnormal laboratory examination results were found. Pregnancy and birth were normal and term. No known family member had shown similar symptoms, including his mother. The urine protein tested by electrophoresis analysis also showed LMW proteins (Table 1), mainly RBP, α1-microglobulin, and β2-microglobulin. However, the urine calcium to creatinine ratio was in the normal range (0.236). Renal biopsy was not performed. Instead, NGS was directly used for screening for Dent disease. A hemizygotic mutation (c. A815G, p.Y272C) was found in the exon of the *CLCN5* gene(ChrX:49850995), which has also been reported in another two Dent 1 case (13, 14). The same mutation was detected in his mother. Two months later, calcium to creatinine ratio was tested in the child showing 0.608 in a spot sample, which also reached the standard of hypercalciuria.

Table 1 outlines the clinical characteristics of the two boys with Dent disease 1. Serum creatinine was measured using an enzymatic method and renal function was calculated using the modified Schwartz formula.

### Renal Biopsy

**Figure 1:**
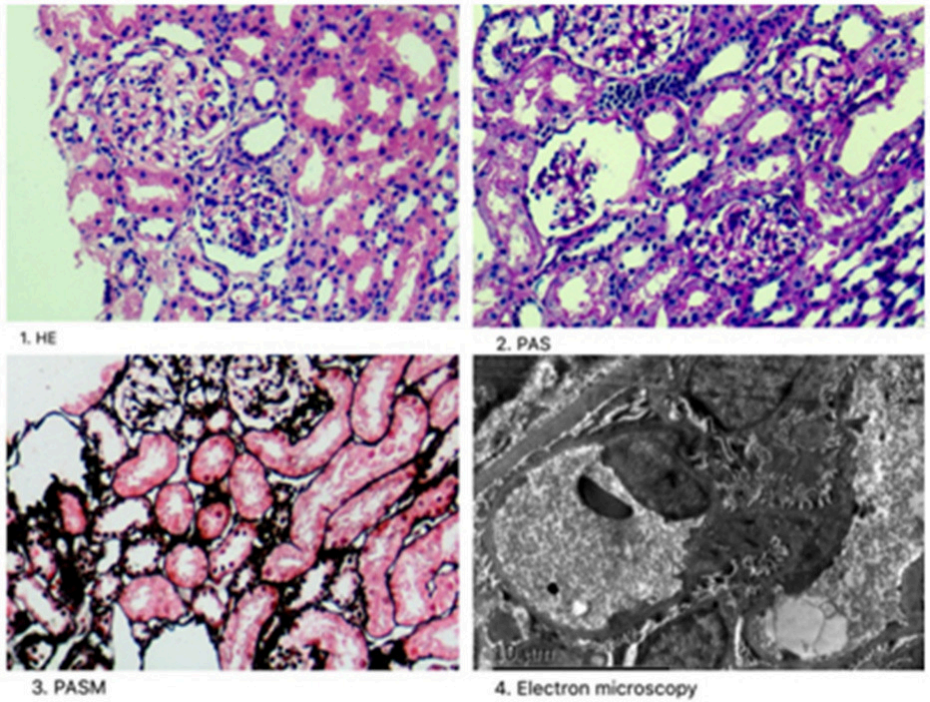
Renal histopathological features in Patient 1. (1) Mesangial cells (2–4 mesangial area) proliferation was shown by hematoxylin-eosin stain (×400); (2) One glomeruli was globally sclerotic shown by Periodic acid–Schiff stain (×400). (3) Inflammatory cell infiltration around the renal interstitium shown by periodic acid-silver metheramine stain; (4) Electron microscopy revealed mild mesangial proliferation with less electron dense deposits.

Renal histopathological features of Patient 1 are displayed in Figure 1. Light microscopy analysis presented with 30 glomeruli. There were areas of FSGS and two of the glomeruli showed global sclerosis. Glomerular mesangial cell proliferation and extracellular matrix hyperplasia were found.Vascular degeneration and protein casts in renal tubules were observed. The renal interstitium showed focal mild edema and inflammatory cell infiltration. Direct immunofluorescent microscopy of the non-sclerotic glomeruli was negative for IgG, IgA, IgM, C3, and C1q staining. Electron microscopy revealed mild mesangial proliferation with less electron dense deposits.

### Genetic Analysis

**Figure 2:**
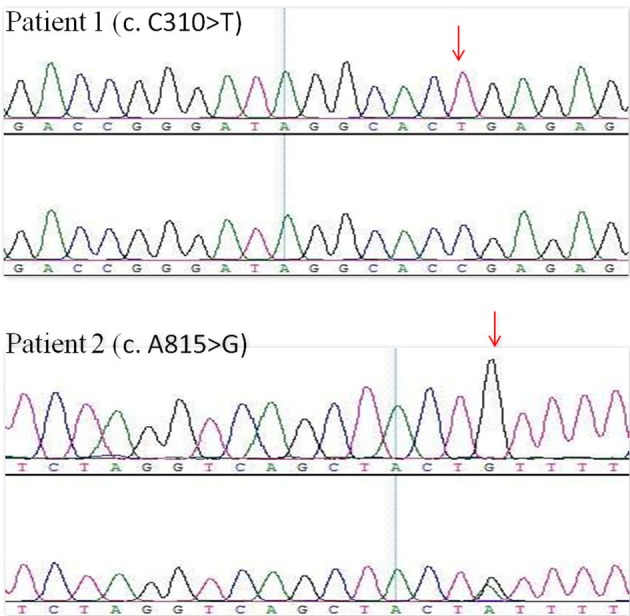
*CLCN5* NGS verification analysis. A hemizygotic mutation (c. C310T,p.R104X) was found in the patient 1. Another hemizygotic mutation (c. A815G,p.Y272C) was found in the patient 2.

Many hereditary renal diseases start with LMW proteinuria, so we did NGS for the two infants. From both patients, 5 ml of whole blood samples were collected in EDTA-anticoagulant tubes. Then, genomic DNA of each patient was extracted using the PUREGENE DNA purification kit from GENTRA using standard protein precipitation procedures. The quality of the DNA was estimated using the Nano-Drop spectrophotometer. Sequencing of all samples was done using the Illumina Nova series platform (Illumina) by Novogene. American College of Medical Genetics and Genomics (ACMG) Standards and Guidelines for the interpretation of sequence variants were followed in this case (15). A hemizygotic mutation (c. C310T,p.R104X) was found in the exon of the CLCN5 gene (ChrX:49834680) in the first child. The mutation is a *de novo* truncated variation (Figure 2, Patient 1) and not included in the 1,000 G databases. Multiple software predict that the mutation is a pathogenic variation and the infant's clinical phenotype is consistent with the phenotype resulting from the genetic mutation (16). In summary, the mutation is a pathogenic variant of Dent disease 1 according to the ACMG Mutation Criteria Guideline (15). The other child was found to possess a hemizygotic mutation (c. A815G,p.Y272C) in the exon of the *CLCN5* gene (ChrX:49850995). His mother with heterozygous mutations (Figure 2, Patient 2) has not shown any related symptoms. This mutation is found to be another pathogenic variant of Dent disease 1 in accordance with the ACMG Mutation Criteria Guideline.

## Discussion

The current study reports the clinical features of two infantile Dent diseases in China. Both cases presented with LMW proteinuria in the nephrotic range, without other main symptoms. Based on the genetic analysis, a diagnosis of Dent disease can be considered.

Proteinuria, especially nephrotic-range proteinuria, is easily confused with other kidney diseases, such as nephrotic syndrome. For patients with proteinuria and normal serum albumin, renal tubular disease should be considered first (8). Urine protein electrophoresis is an effective way for finding LMW proteinuria (17), which shows as excessive urinary loss of α1-microglobulin, β2-microglobulin, or other LMW plasma proteins. Zhang et al. (18, 19) examined the ratio of urinary α1-microglobulin to microalbumin in 24 Chinese pediatric patients with renal tubular and interstitial diseases, suggesting that a ratio of urinary α1-microglobulin to microalbumin >1 could be used as a diagnostic criterion for tubuloproteinuria. In both our cases, the ratio of urinary α1-microglobulin to microalbumin tested multiple times were all >1, which may have some benefits for early diagnosis.

Hypercalciuria always appears relatively later than LMW proteinuria. In a large Dent disease cohort observed by Blanchard et al. (20), hypercalciuria with decreased glomerular filtration was absent in 40% of the patients under 30 years and 85% of those over the age of 30 years. The methods to diagnose hypercalciuria do not have a standardized criteria (21, 22), which may be a cause for the misdiagnosis of hypercalciuria. In Blanchard's article (20), ESRD occurred at a median age of 40 years. Therefore, even if there is no hypercalciuria, we should also consider Dent disease 1 as a differential diagnosis.

FSGS, discovered in renal histopathology of the first case, may have potential links with Dent disease 1 (5). Valina (23) reported a case of a 5-year-old boy who presented with asymptomatic nephrotic-range proteinuria and was later diagnosed with Dent disease. His renal biopsy also showed FSGS. Clinical renal pathology reports and slides collected from 30 patients in 8 countries found that 83% had focal global glomerulosclerosis (24). Thus, FSGS is likely a characteristic of Dent disease 1, though the mechanism is not clear.

Extremely high LMW proteinuria and absence of history or clinical data, indicating renal diseases that cause proximal tubular dysfunction, can be a diagnostic criteria for Dent disease (25), which accounts for only 8% of renal failure in Japanese patients, whereas in Europe and the USA, renal insufficiency develops after middle age. Early diagnosis decreases the improper uses of corticosteroids and immunosuppressive therapy and may slow down the progression of renal insufficiency (25), which shows that early diagnosis of Dent disease 1 is critical for the prognosis, even though it presents with atypical symptoms at onset.

Sequencing is particularly effective for the early diagnosis of the ambiguous Dent disease 1 (13, 26, 27), especially NGS. Mansour-Hendili et al. (16) reviewed the published mutations in the *CLCN5* gene, as well as their phenotype and 42 previously undescribed mutations, showing that NGS is being used with increasing frequency. However, the association between phenotype and genotype of Dent disease 1 has not been fully recognized because its symptoms can vary even within the same mutation loci (28, 29). Defined by the ACMG Mutation Criteria Guideline, *CLCN5* mutation can be seen as a pathogenic variant of Dent disease 1 and can provide strong evidence for early diagnosis, together with LMW proteinuria.

## Concluding Remarks

Overall, there are no clear diagnostic criteria for Dent disease 1. LMW proteinuria, as an essential symptom, may be found with the onset of nephrotic-range proteinuria. Urine protein electrophoresis is an effective way of detecting LMW proteinuria. Renal histopathology shows that FSGS may have potential links with Dent disease 1. Pathogenic *CLCN5* mutations detected by genetic analysis, as well as LMW proteinuria, can be used as criteria for the early diagnosis of Dent disease 1.

## Author's Note

Written informed consent has been obtained from the parents of the two patients for publication of this case report.

## Ethics Statement

This study was carried out in accordance with the recommendations of the second xiangya hospital of central south university with written informed consent from all subjects.

## Author Contributions

All authors listed have made a substantial, direct and intellectual contribution to the work, and approved it for publication.

### Conflict of Interest Statement

The authors declare that the research was conducted in the absence of any commercial or financial relationships that could be construed as a potential conflict of interest.
